# Clinical Improvement and Prolonged Survival With High Dose Intrathecal Methotrexate for Carcinomatous Meningitis Secondary to a Lung Adenocarcinoma

**DOI:** 10.4021/wjon358w

**Published:** 2011-10-28

**Authors:** Florian Clatot, Philippe David, Sophie Laberge-Le-Couteulx, Emmanuel Blot

**Affiliations:** a Department of Medical Oncology, Centre Henri Becquerel, Rouen, France; b Department of Pneumology, CHI Les Feugrais, Elbeuf, France; c Department of Pathology, Centre Henri Becquerel, Rouen, France; d Department of Medical Oncology, Centre Saint Yves and CH Bretagne Atlantique, Vannes, France

**Keywords:** Lung adenocarcinoma, Carcinomatous meningitis, Leptomeningeal metastasis, Intrathecal, Methotrexate

## Abstract

Carcinomatous meningitis (CM) is a devastating complication of advanced cancers. No standard treatment is available for CM secondary to solid tumors. In particular, very few data have been published for CM related to lung adenocarcinoma. Herein we report the case of a 39-year old woman treated for a metastatic lung adenocarcinoma, without EGFR mutation, complicated by a CM. Intrathecal administrations of high dose methotrexate produced both a clinical improvement of symptoms related to CM and an unusually delayed survival.

## Introduction

Carcinomatous meningitis (CM) arises in about 5% to 8% of advanced solid cancers [1]. This complication is associated with a very poor prognosis of few weeks, especially when the primitive cancer is the lung [2]. Treatment options may include systemic chemotherapy, intrathecal chemotherapy or external radiation therapy [1]. Among intrathecal chemotherapy, only 3 molecules can be used: ThioTEPA, cytarabine and methotrexate [1]. Cytarabine treatment is usually given twice a week [1, 3] or twice a month using a sustained-release formulation (DepoCyt®) [4]. Methotrexate is mainly used with 10-20 mg administrations twice a week. In breast cancer related CM, the use of high dose intrathecal methotrexate (15 mg for 5 days every two weeks) was suggested to improve survival [5]. This interesting result in breast-related CM was further confirmed in two retrospective studies [6, 7].

The lack of consensus for treatment of CM in solid tumors results from the few number of randomised studies. Furthermore, published studies often do not differentiate the primary tumour, although they can have different sensitivity to chemotherapy [1, 2].

For CM related to lung adenocarcinoma (CMLA), very few reports are available in the literature. Herein we present a case of CMLA treated with high dose intrathecal methotrexate.

## Case Report

A 39-year old woman, with a medical history of 20 pack-years of smoking, was diagnosed lung adenocarcinoma with liver metastasis in September 2009. Molecular analysis of the tumor revealed no EGFR mutation. She was treated with cycles of permetrexed (500 mg/m^2^) and cisplatinum (75 mg/m^2^) every 21 days from September 2009 to January 2010, with a stable disease on CT scan evaluation after 6 cycles of chemotherapy.

In January 2010, she had a persistent headache associated with photophobia and a progressive decrease of her performance status. Radiological explorations included a CT and a MRI of the brain and were considered normal. A cerebrospinal fluid (CSF) exploration was then performed, revealing the presence of carcinomatous cells, compatible with an adecarcinoma of the lung. The CSF protein level was slightly increased (0.47 g/L), whereas CSF glucose level was normal. Intrathecal DepoCyt® (50 mg) was delivered the 17th of February 2010, followed by marked aggravation of clinical symptoms and worsened performance status, despite oral prednisone intake at 1 mg/kg.

The patient was then treated with intrathecal injections of methotrexate (15 mg, day 1-5) and prednisolone 30 mg (day 1) with oral folinic acid rescue (25 mg 12 h after methotrexate, day 1-5) [6]. A cycle was performed every 2 weeks with repeated lumbar punctures. Four cycles of high dose methotrexate were administered between March and April. A clinical improvement was noticed after the first cycle of chemotherapy, with decrease in headaches, improvement of performance status, allowing the diminution of prednisone intake. Clearance of carcinomatous cells was observed on CSF exploration after the second cycle of chemotherapy and confirmed until the end of intrathecal injections. Treatment was well tolerated and no clinical or biological toxicity was noticed. Interruption of intrathecal treatment was decided with regards to clinical and cytological response after four intrathecal treatment injections.

At the end of intrathecal treatment, CT scan evaluation of lung and liver disease showed stability of systemic disease, and systemic maintenance chemotherapy by permetrexed (500 mg/m^2^ every 21 days) was then performed. Lung disease progression was noted in September 2010, associated with reappraisal of CM symptoms, and rapid decrease of performance status, leading to chemotherapy interruption.

Death occurred in October 2010, 13 months after the diagnosis of the cancer, and 35 weeks after the cytological diagnosis of CM.

## Discussion

Even if no randomized studies have been dedicated to CMLA, available data underline the difficulty to manage this kind of complication.

Among randomised published studies, Hichins et al in 1987 treated only 3 non small cell lung carcinoma (NSCLC) [8]. Grossman et al in 1993 reported a treatment of 12 lung cancer (without distinction between SCLC and NSCLC), with a median survival of 8 weeks after intrathecal treatment by methotrexate (10 mg twice a week) or ThioTEPA (10 mg twice a week) [9]. Glantz et al treated 6 NSCLC by either intrathecal DepoCyt® (50 mg every two weeks) or methotrexate (10 mg twice a week), with only one “response” (CSF clearance and neurological stabilization or improvement), and without data on survival [4]. Kim et al in 2003 provided the largest randomised study including lung adenocarcinoma. Twenty five patients with CMLA were treated twice a week by intrathecal methotrexate (15 mg) with or without hydrocortisone (15 mg/m^2^) and cytarabine (30 mg/m^2^). Median overall survival in the methotrexate group was 10.4 weeks, and the longest survival in this cohort was 29 weeks. Neurological response (stabilization or improvement for at least 4 weeks) was observed for half of patients who were not treated by radiation therapy. But the median neurological response was only 5.1 weeks [3].

Chamberlain et al published in 1998 a prospective but non-randomised study on CM in lung, with 24 adenocarcinomas included. Treatment was based on initial intrathecal methotrexate (2 mg for 5 days every 2 weeks) followed by intrathecal cytarabine and even ThioTEPA in case of progression of the disease. In this study, the median overall survival was 5 months (max 12 months) [10].

Among retrospective studies, Chamberlain et al reported in 2009 the treatment of 8 NSCLC (without distinction of the histological subtype). The median survival among those 8 patients was 6 weeks, with a longest survival of 17 weeks [11]. Other studies have reported comparable results [2, 12]. Finally, a potential benefit of targeted therapies has recently been reported in CM related to EGFR-mutated lung adenocarcinoma [13].

To our knowledge, treatment by CMLA with high dose intrathecal methotrexate has not previously been reported. Data published on breast-related CM showed that this treatment is easily administrated by lumbar puncture (without implantation of an Ommaya device), and efficient with few side-effects [5-7].

### Conclusion

Even if managing CM is still a challenge and remains a palliative approach, the present case-report suggests that high-dose intrathecal methotrexate can provide major clinical improvement, cytological CSF response and unusual survival duration with no deleterious side-effect.
